# Supplementary material for: Developmental basis of SHH medulloblastoma heterogeneity
Source: Nat Commun. 2024 Jan 8;15:270. doi: 10.1038/s41467-023-44300-0 (PMC10774283; doi:10.1038/s41467-023-44300-0)
Supplement: Supplementary file 1 — Supplementary Information [file 41467_2023_44300_MOESM1_ESM.pdf]

a

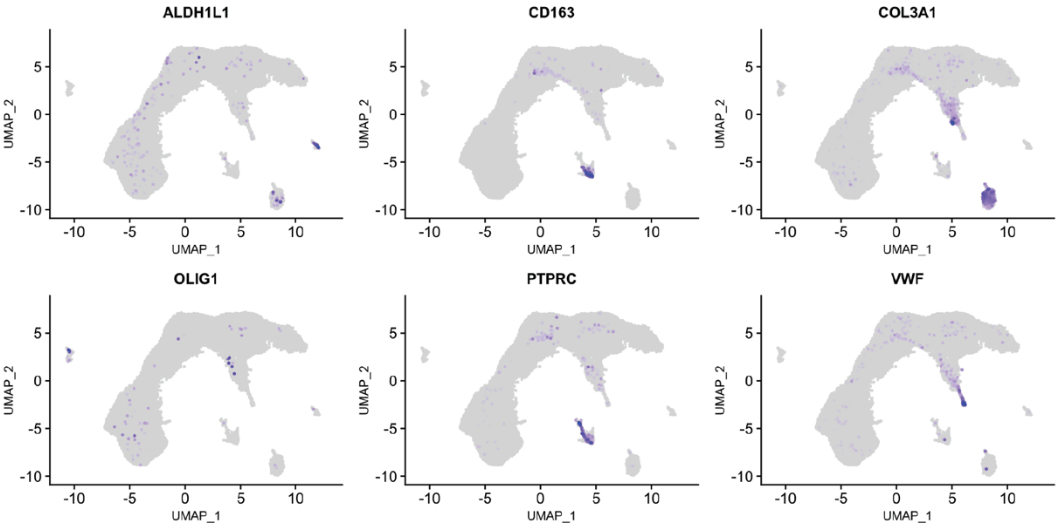

b

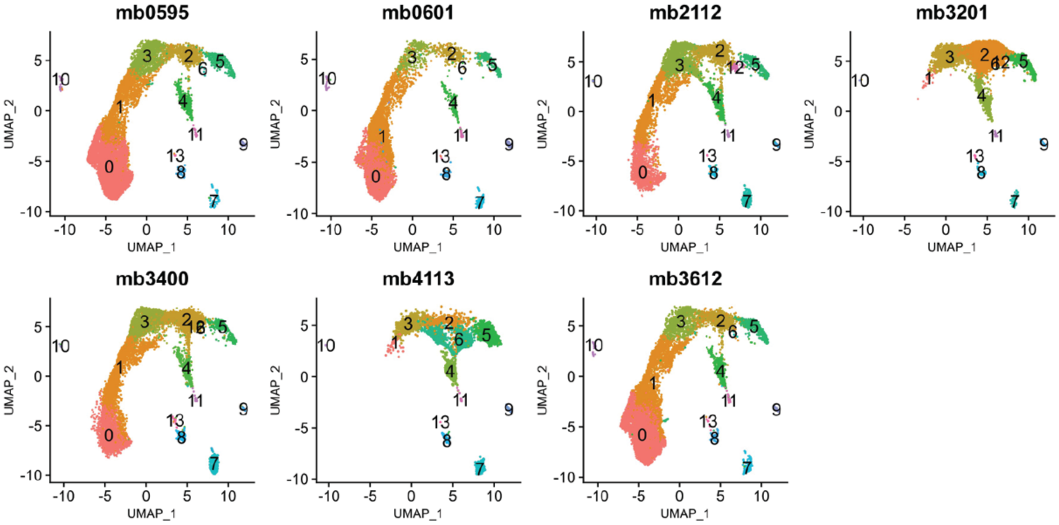

<u>Supplementary Figure 1) Summary of MBEN snRNA-seq</u>

A) <u>Marker Genes for Non-Malignant Cell Types:</u> UMAP plots show integrated snRNA-seq data from all MBEN tumors. Astrocytes (ALDH1L1), Endothelial cells (COL3A1), Immune cells (PTPRC/CD45), Macrophages (CD163), Microglia (VWF), and Oligodendrocytes (OLIG1). Expression minimum is 70th percentile for each marker.

B) <u>Cell Type Heterogeneity in MBEN tumors</u>: Clusters 0, 1, 2, 3, 4, 5, 6 and 12 represent malignant nuclei. Cluster 6 is almost exclusively present in MB4113 and almost all of cluster 12 is from MB2112. Clusters 2, 3, 4, and 5 appear in all samples, while clusters 0 and 1 are found in most tumors.

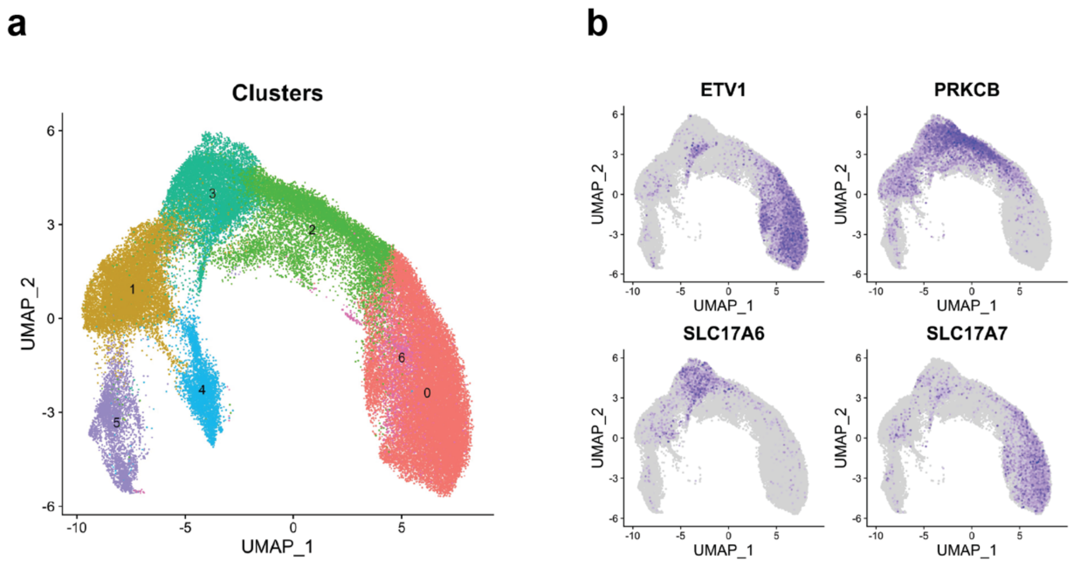

<u>Supplementary Figure 2) snRNA-seq Clusters and Marker Genes</u>
A) <u>Re-clustering of Malignant Cells:</u> UMAP plot of malignant cells after reprocessing and another round of clustering. Every cluster besides 4 corresponds to a stage of GN development. Cluster 4 is high in ribosomal genes and excluded from analyses related to GN development. Cluster 6 was combined with Cluster 0 given the close proximity in UMAP space.
B) <u>Expression of Additional GN Development Markers:</u> ETV1 is a transcription factor that plays a crucial role in GN maturation and GRIN2C expression. PRKCB is a marker of calcium signaling, which drives GN migration. SLC17A6 (VGLUT2) is expressed early in GN development but is replaced by SLC17A7 (VGLUT1) as GN reach the IGL and mature. Expression minimum is 80th percentile for each marker.

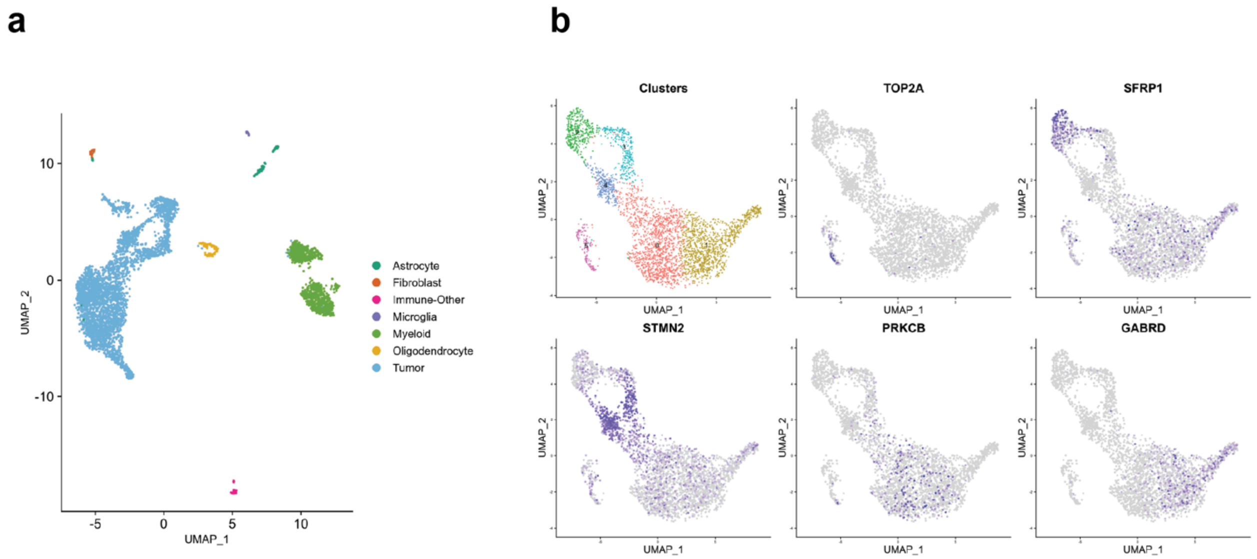

<u>Supplementary Figure 3) Summary of scRNA-seq Data (BT2019110)</u>
A) <u>UMAP Plot for Single-Cell RNA-Seq of MBEN Tumor:</u> Malignant and non-malignant cell types from sample BT2019110 are labeled in the legend.
B) <u>GN Development Genes in MBEN scRNA-Seq:</u> Expression profiles for key genes related to GN development and SHH MB in BT2019110.

Dataset	Subtypes / Cell Types	Assay Type	Source Paper	Description
Archer Proteomics	SHHa, SHHb	Bulk Proteomics	Archer *et al.* 2018	Proteomic subtypes of SHH MB that are not obvious in RNA or methylation data
Consensus SHH	SHH-1, SHH-2, SHH-3, SHH-4	Bulk Transcriptomics	Cavalli *et al.* 2017	Consensus subtypes of SHH MB that were first defined by clustering of methylation and RNA data
Human Cerebellum	GCP, GN-diff1, GN-diff2, GN-defined	Single-Nuclues RNA-Seq	Okonechnikov *et al.* 2023	Cell types from stages of granule neuron development from snRNA-Seq from cerebella of developing human brains
Korshunov MBEN	TCL1, TCL2	Bulk Transcriptomics	Korshunov *et al.* 2020	Transcriptomic subtypes of MBEN tumors with clear outcome differences
MBEN scRNA-Seq	Clusters 0,1,2,3,4,5	Single-Cell RNA-Seq	This work	Cell types from scRNA-Seq of one MBEN tumor
MBEN snRNA-Seq	GCP-Cyc, GCP, GN-premig, GN-mig, GN-postmig, Ribosomal	Single-Nuclues RNA-Seq	This work	Cell types from snRNA-Seq of seven SHH MBs with MBEN histology
P14 Mouse Cerebellum	GCP-Cyc, GCP, GN-premig, GN-mig, GN-postmig	Single-Cell RNA-Seq	Vladiou *et al.* 2019	Cell types for stages of granule neuron development from scRNA-Seq from cerebella of P14 mouse brain
Riemondy	SHH-A1, SHH-A2, SHH-B1, SHH-B2, SHH-C1, SHH-C2	Single-Cell RNA-Seq	Riemondy *et al.* 2022	Cell types from scRNA-Seq of 9 pediatric SHH MBs from SHH-1, SHH-3, and SHH-4 subtypes

Supplementary Figure 4) Summary of Cell Types and Molecular Subtypes

Table describes the datasets and associated manuscripts used for each study included in Figure 2B: Archer proteomic subtypes [1], Consensus SHH MB subtypes [2], Korshunov MBEN transcriptional subtypes [3], human GN development [4], MBEN snRNA-seq, MBEN scRNA-seq, P14 Mouse scRNA-seq [5] and Riemondy SHH MB scRNA-seq [6].

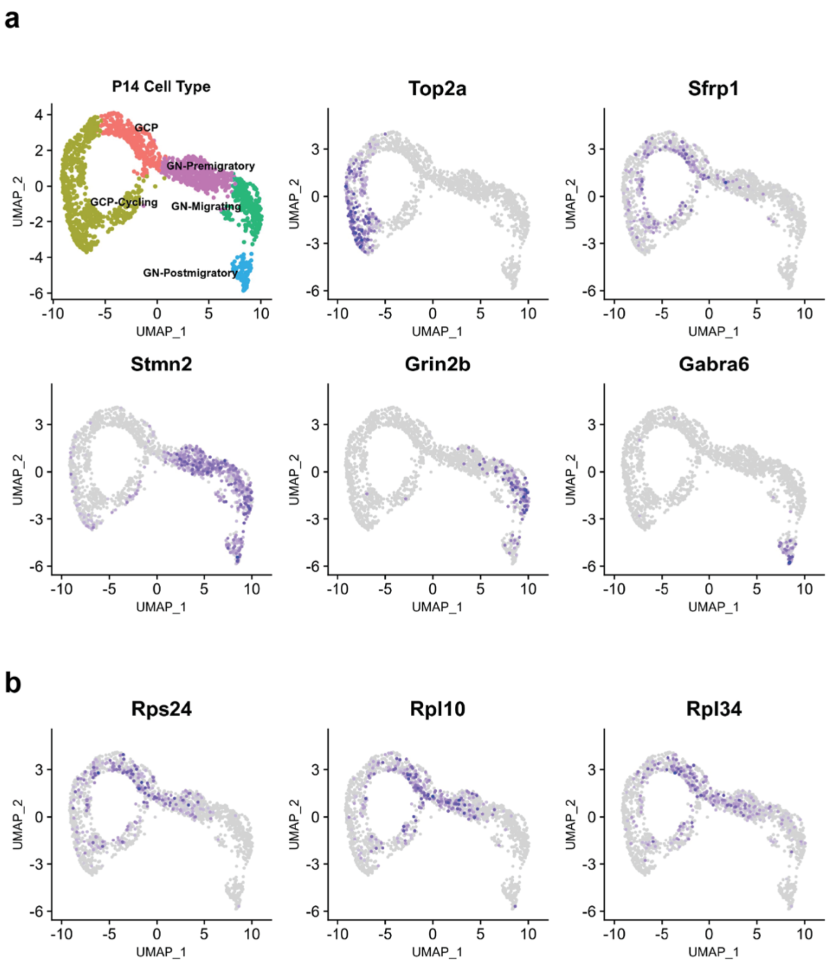

<u>Supplementary Figure 5) Summary of Mouse scRNA-Seq Data (Vladoiu *et al.* [5])</u>

A) <u>Cell Type Heterogeneity in P14 Mice Granule Neurons</u>: Top left panel shows annotations for specific cell types. The other panels show markers of each cell stage.

B) <u>Ribosomal Gene Expression:</u> There are a subset of cells that express high levels of ribosomal markers like Rps24, Rpl10, and Rpl34. This may correspond to the ribosomal cluster identified during human MB snRNA-seq (Supplementary Figure 2).

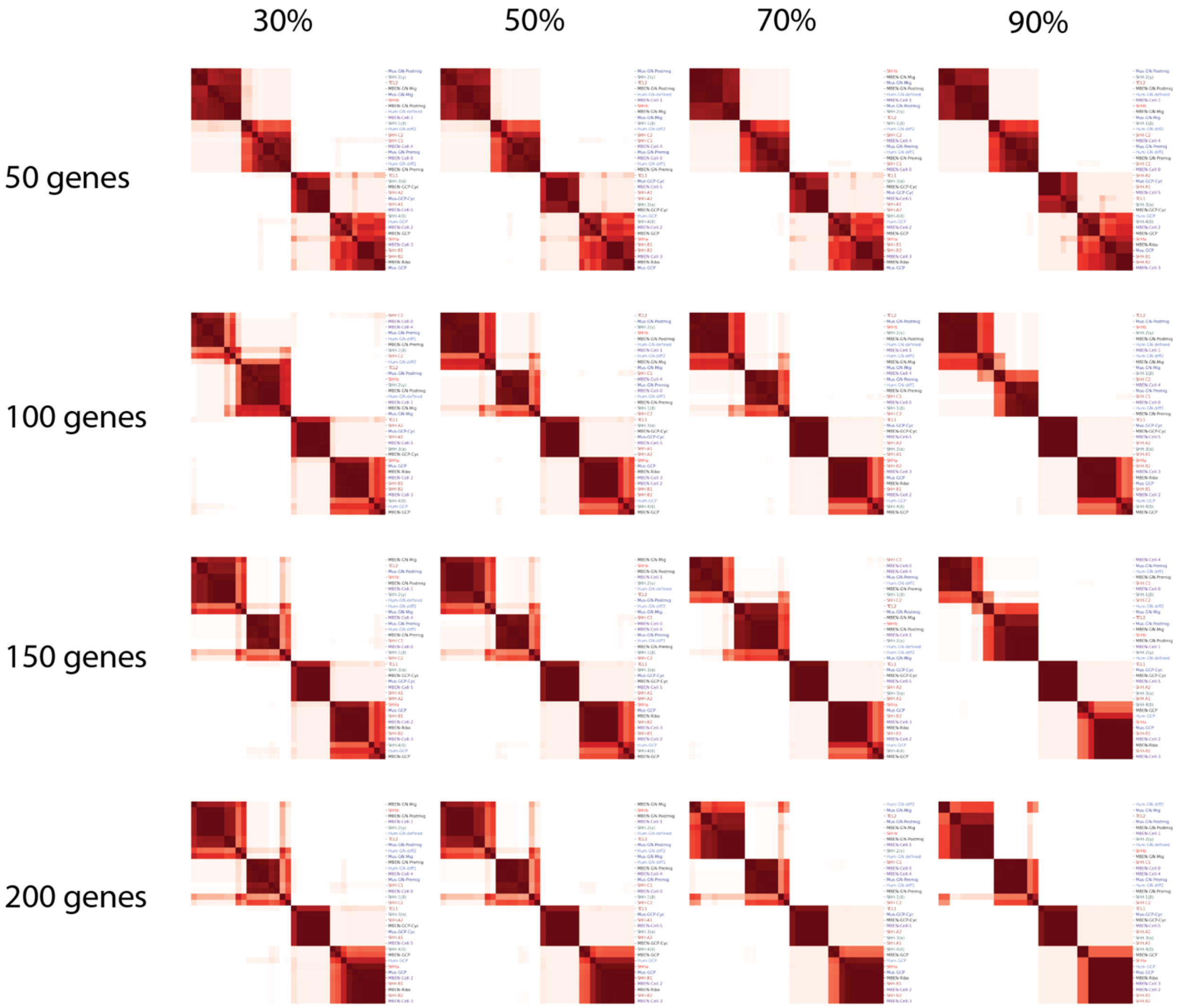

<u>Supplementary Figure 6) Consensus Cluster Parameter Sweep</u>
The left axis indicates the number marker genes included. The top axis shows the percentage of SHH tumors sampled for each clustering. The exact co-clustering differs across parameters, but the high-level trends are consistent for all gene set sizes and sampling percentages.

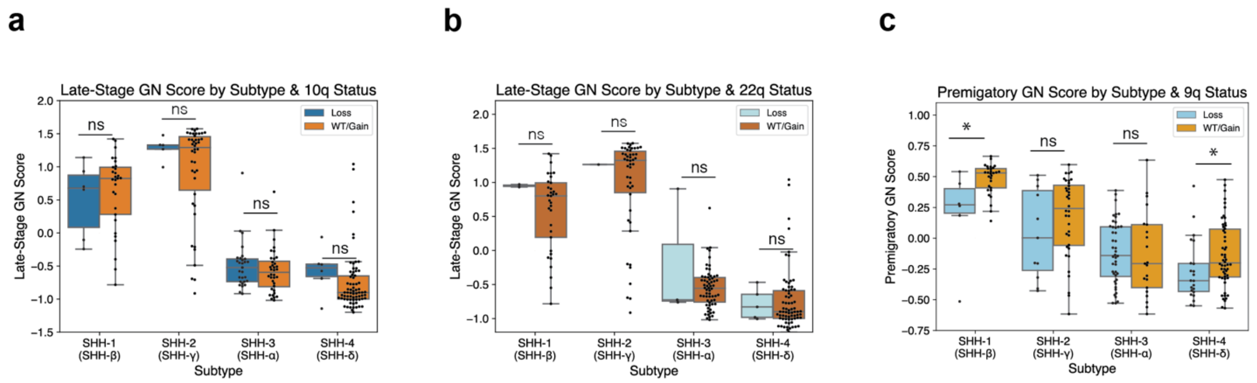

Supplementary Figure 7) Genomic Trends by Consensus Subtype

A) Late-Stage GN Score by 10q Status: Y axis is late-stage GN score (migrating GN + postmigratory GN). X axis is subtype (SHH-1, SHH-2, SHH-3, or SHH-4) further divided by 10q status. There are no significant differences in late-stage GN score for 10q. The boxplots from left to right represent the following number of samples: 6, 29, 5, 42, 29, 36, 6, and 70.

B) Late-Stage GN Score by 22q Status: Y axis is late-stage GN score. X axis is subtype further divided by 22q status. There are no significant differences in late-stage GN score for 22q. The boxplots from left to right represent the following number of samples: 2, 33, 1, 46, 3, 62, 5, and 71.

C) Premigratory GN Score by 9q Status: Y axis is premigratory GN score. X axis is subtype further divided by 9q status. Premigratory GN shows different trend with chromosome 9q than late-stage GNs do. For example, the difference in SHH-2 samples is less pronounced for the premigratory GN compared to the late-stage GN (Figure 3D). Single asterisk * indicates significant difference (two-sided Mann-Whitney p-value < 0.05) for SHH-1 and SHH-4. The boxplots from left to right represent the following number of samples: 6, 29, 11, 36, 44, 21, 22, 54.

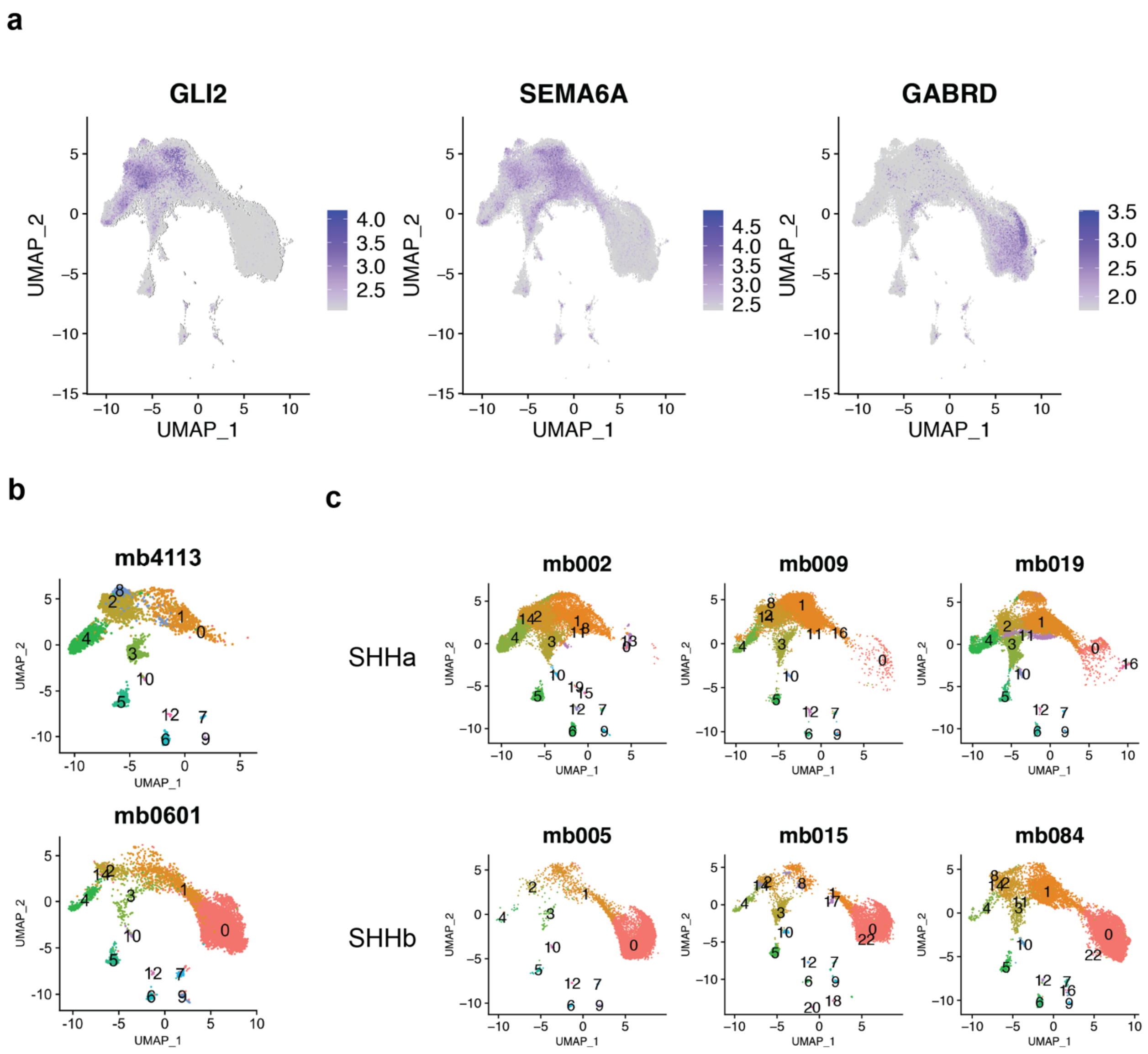

<u>Supplementary Figure 8) snRNA-seq Validation Cohort</u>

A) <u>Validation Cohort Marker Genes</u>: marker gene expression for GLI2, SEMA6A, and GABRD that indicate GCPs, premigratory GNs, and late-stage GNs respectively. Each marker is showing signal above the 80th percentile to highlight cells with the highest expression.

B) <u>UMAP for MBEN Tumors:</u> UMAP plots for two representative tumors in new integrated cohort. MB4113 shows no evidence of late-stage GNs, while MB0601 does (cluster 0).

C) <u>UMAP for Validation Cohort:</u> UMAP plots for six tumors. Top row (MB002, MB009, and MB019) are tumors with the SHHa proteomic subtype and are primarily composed of GLI2+ cells and SEMA6A+ cells. The bottom row (MB005, MB015, and MB084) are samples called as SHHb proteomic subtype and they each contain a substantial proportion of GABRD+ cells.

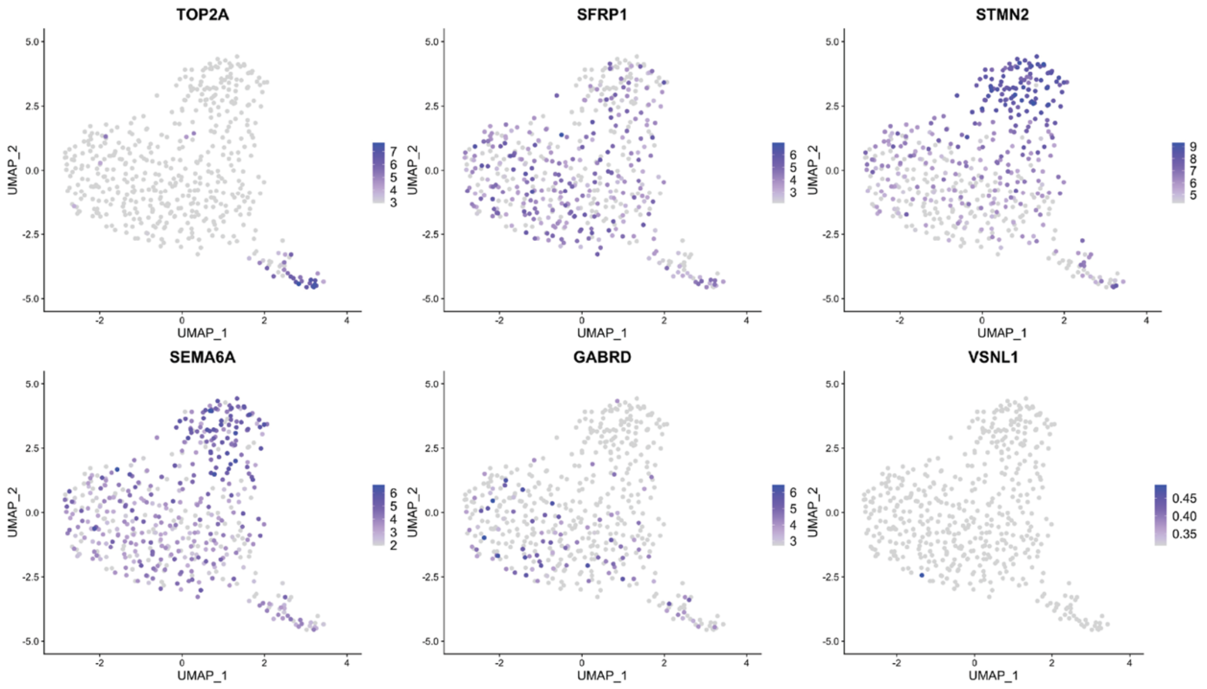

Supplementary Figure 9) Feature Plots for SJ557 from Hovestadt *et al.*[7]
Each individual chart shows the same UMAP plot, with color scale marking the relative expression for the indicated gene (TOP2A, SFRP1, STMN2, SEMA6A, GABRD, or VSNL1).

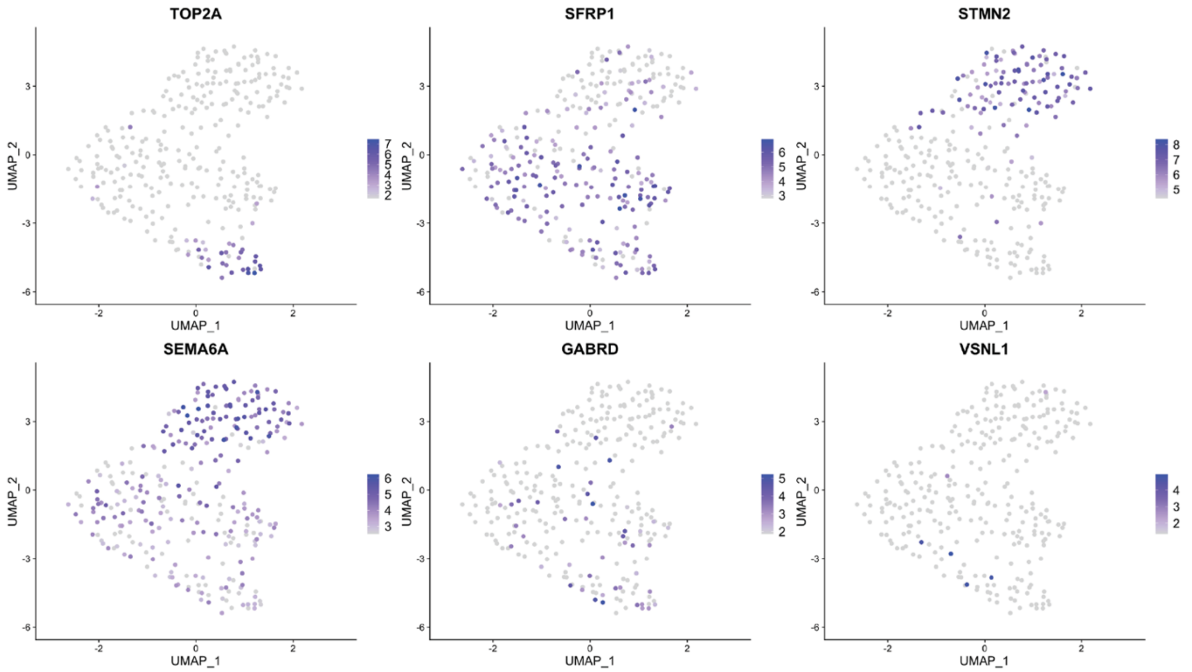

Supplementary Figure 10) Feature Plots for SJ454 from Hovestadt *et al.*[7]
Each individual chart shows the same UMAP plot, with color scale marking the relative expression for the indicated gene (TOP2A, SFRP1, STMN2, SEMA6A, GABRD, or VSNL1).

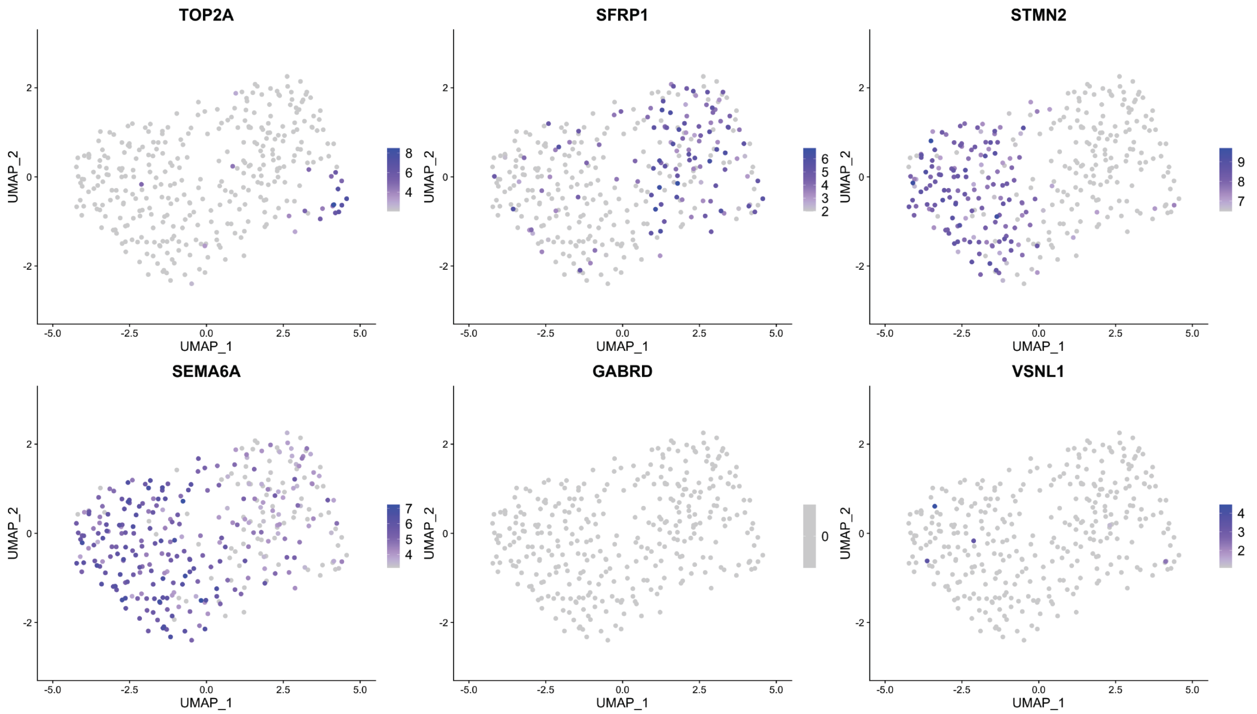

Supplementary Figure 11) Feature Plots for MUV41 from Hovestadt *et al.* [7]

Each individual chart shows the same UMAP plot, with color scale marking the relative expression for the indicated gene (TOP2A, SFRP1, STMN2, SEMA6A, GABRD, or VSNL1).

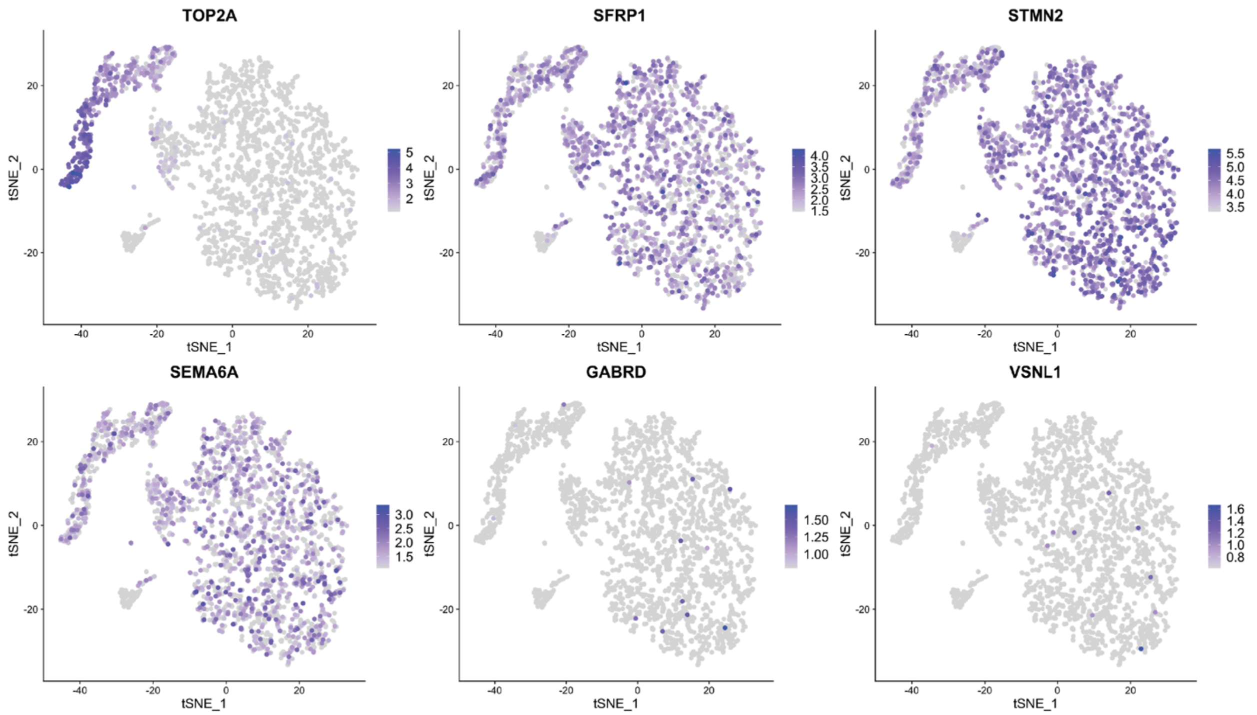

Supplementary Figure 12) Feature Plots for BT2017017 from Vladoiu *et al.* [5]

Each individual chart shows the same t-SNE plot, with color scale marking the relative expression for the indicated gene (TOP2A, SFRP1, STMN2, SEMA6A, GABRD, or VSNL1).

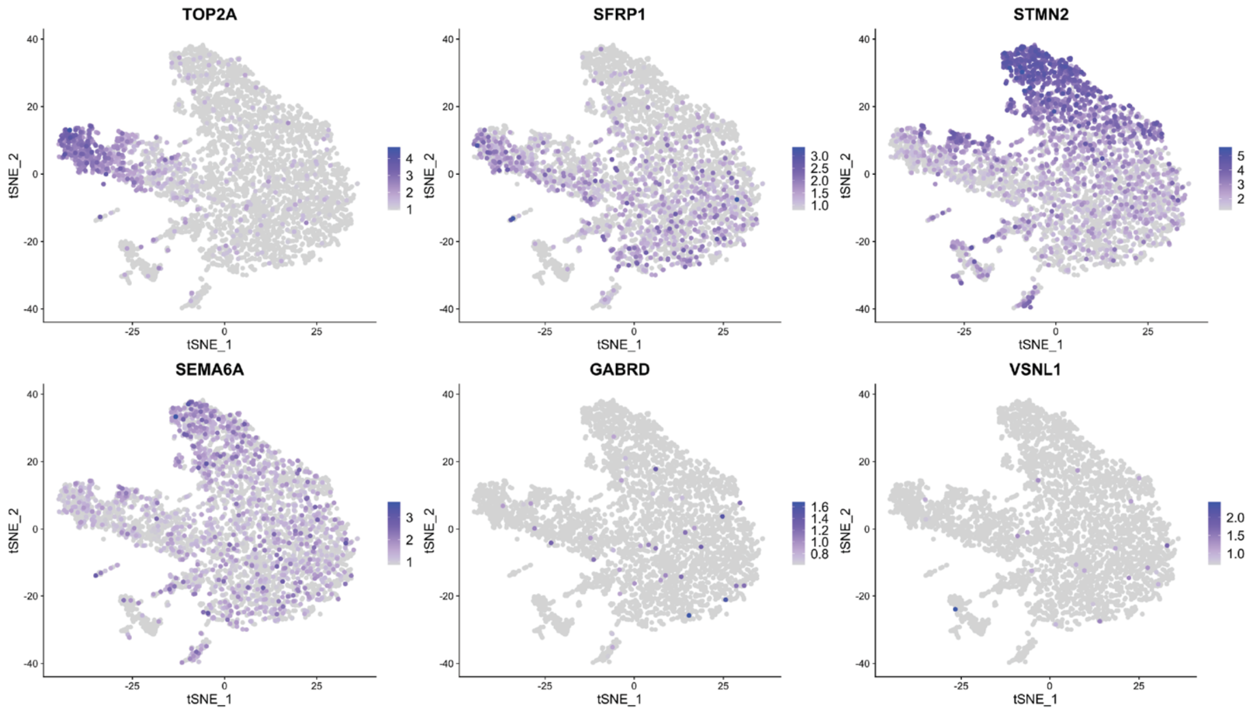

<u>Supplementary Figure 13) Feature Plots for SM4217 from Vladoiu *et al.* [5]</u>
Each individual chart shows the same t-SNE plot, with color scale marking the relative expression for the indicated gene (TOP2A, SFRP1, STMN2, SEMA6A, GABRD, or VSNL1).

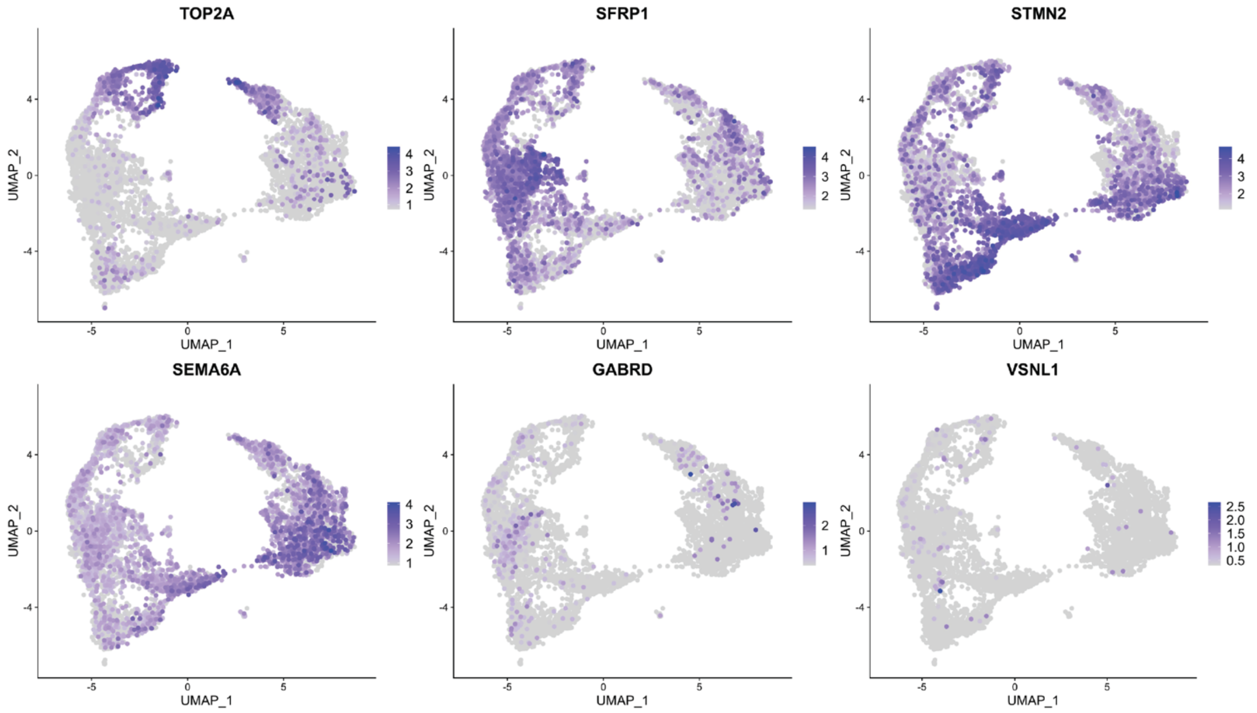

<u>Supplementary Figure 14) Feature Plots for Riemondy *et al.* [6]</u>
Each individual chart shows the same UMAP plot, with color scale marking the relative expression for the indicated gene (TOP2A, SFRP1, STMN2, SEMA6A, GABRD, or VSNL1). Each plot contains data from the 9 SHH tumors from the Riemondy *et al.* study.

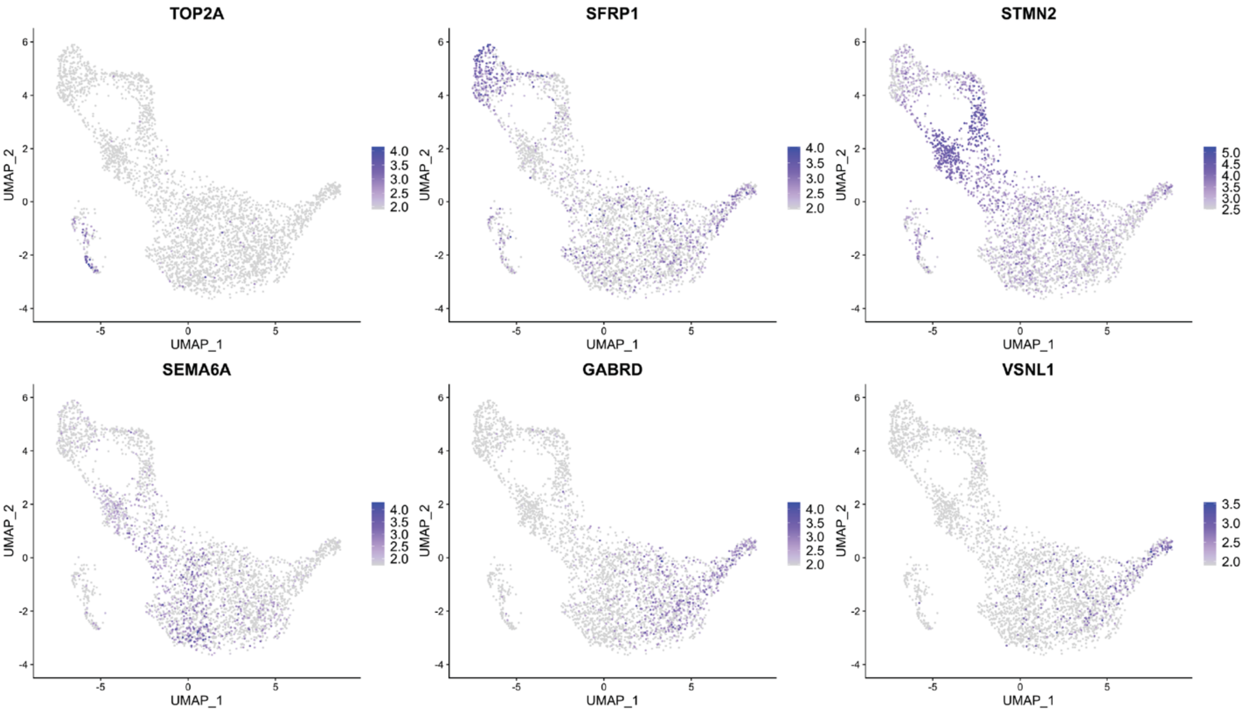

Supplementary Figure 15) Feature Plots for MBEN tumor BT2019110

Each individual chart shows the same UMAP plot, with color scale marking the relative expression for the indicated gene (TOP2A, SFRP1, STMN2, SEMA6A, GABRD, or VSNL1). BT20191110 is the one MBEN tumor with scRNA-seq data from this study and the tumor contains GABRD+/VSNL1+ cells not observed in the other scRNA-seq samples (Supplementary Figures 9-14).

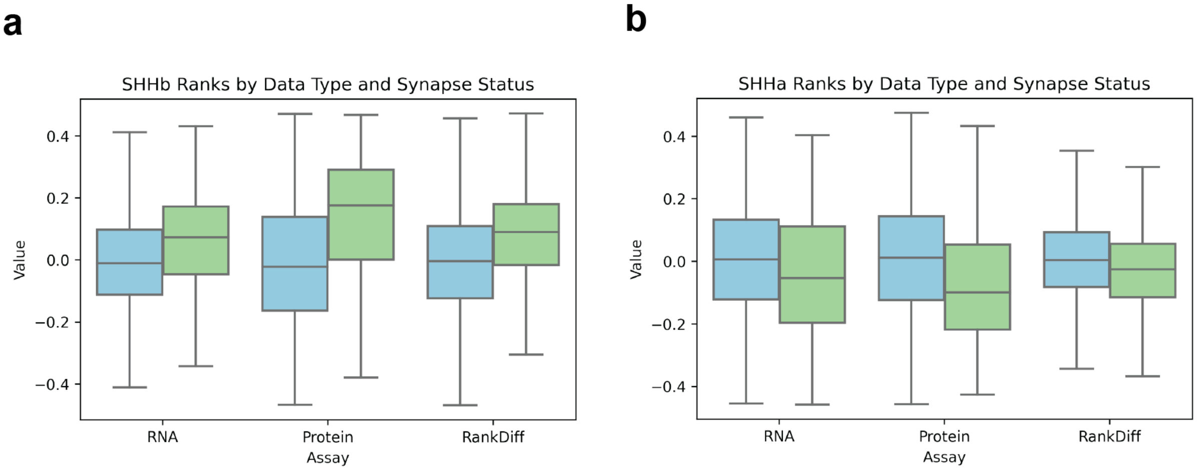

Supplementary Figure 16) SHHa and SHHb Gene Ranks by Synapse Status

X axis is the specific assay being analyzed. Y axis is rank for that assay. Data is split between non-synaptic genes (blue on left, 7846 genes) and synaptic genes (green on right, 828 genes). For plot A, each row in the boxplot represents the mean value for a gene across all SHHb tumors. For plot B, each row represents mean value for a gene across all SHHa tumors. Each boxplot shows data quartiles, excluding outliers beyond 1.5 x IQR.

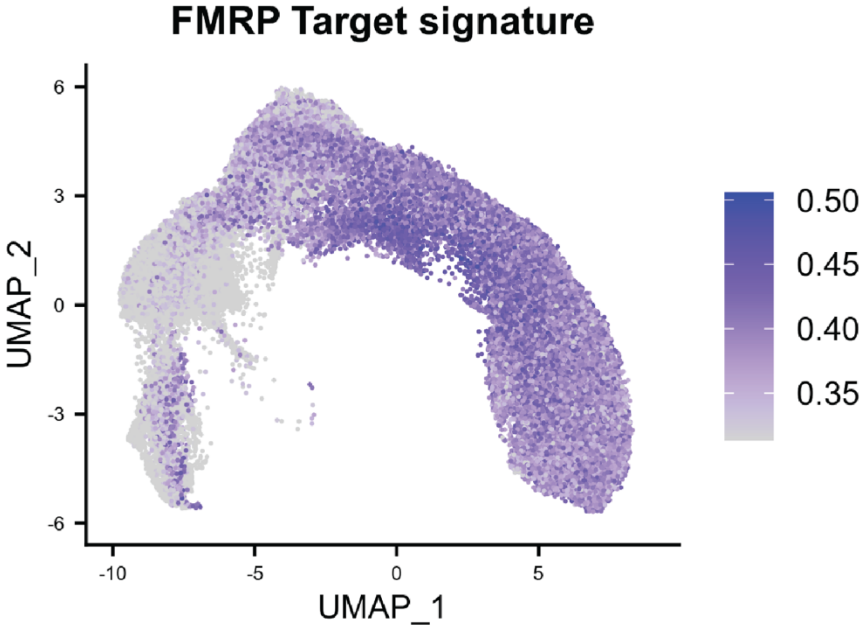

Supplementary Figure 17) FMRP Target Genes in snRNA-seq Data
UMAP plot showing gene set enrichment for FMRP target genes [8]. Plot shows scores from 0.30 to 0.50 to highlight the strongest signal occurring in the tumor cells resembling late-stage GNs.

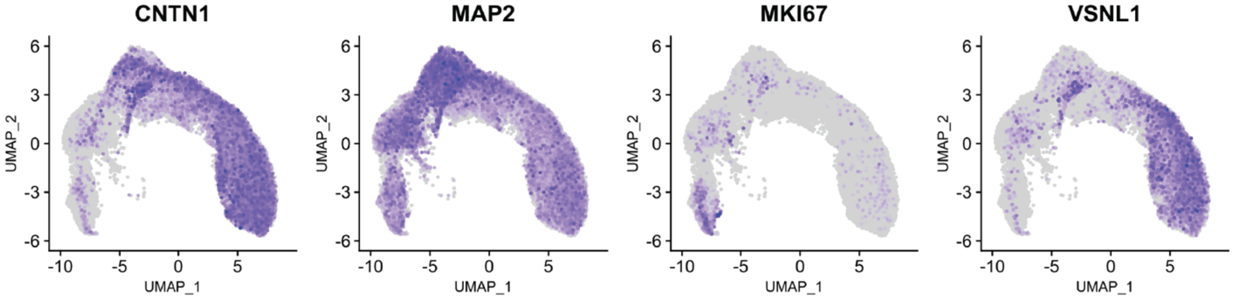

Supplementary Figure 18) Marker Gene Expression for mIHC Markers
snRNA-Seq expression for CNTN1, MAP2, MKI67, and VNSL1 for MBEN tumors included in Figure 1D. Feature maps only show expression above 80th percentile to highlight nuclei with highest values. VSNL1 shows much stronger expression for tumor cells mimicking late-stage GNs than tumor cells resembling GCPs or premigratory GNs.

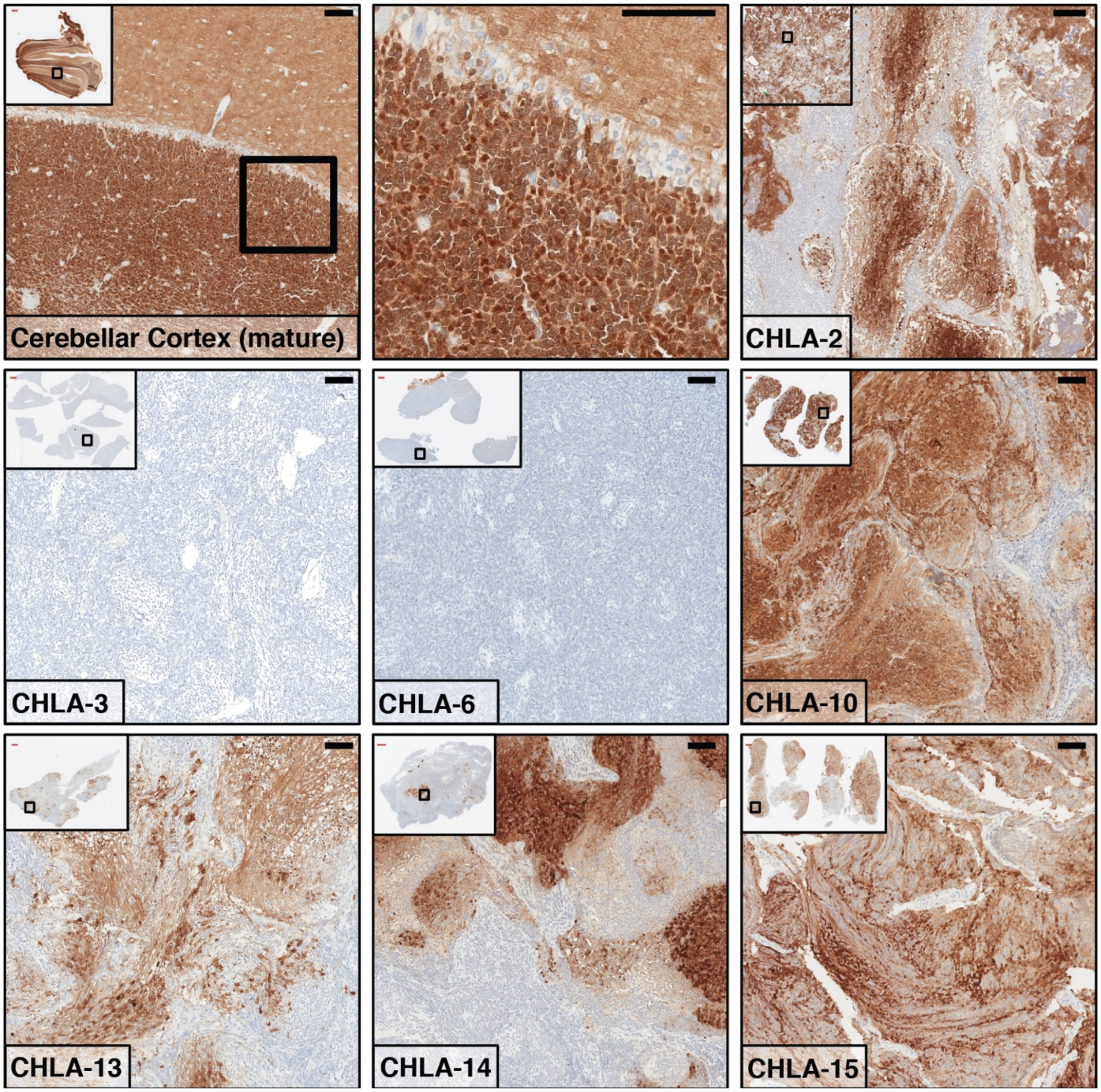

<u>Supplementary Figure 19) VSNL1 Staining from Nodular CHLA Samples</u>
The top left section shows VSNL1 staining in the healthy cerebellum and the top center section shows a zoomed in version of a section overlapping the internal granule layer, Purkinje layer, and molecular layer. The other panels highlight the heterogeneity of VSNL1 staining in pediatric SHH MBs. In total, there are two examples of healthy cerebellum from one patient and seven tumor sections from six unique MB patients. CHLA-2 and CHLA-10 are distinct sections from the same patient. Scale bars at the top right of each panel indicate 100 μm.

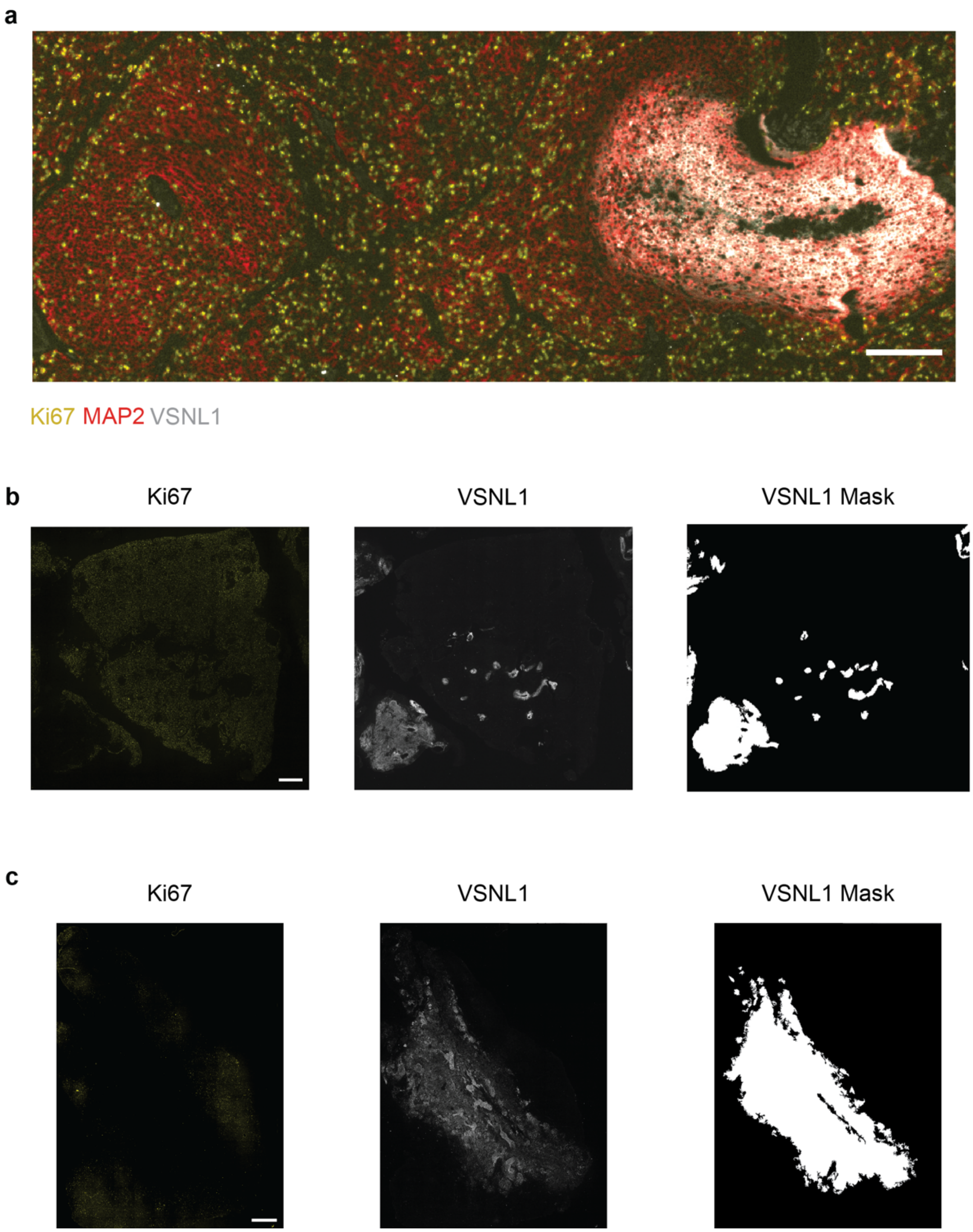

Supplementary Figure 20) Ki67 and VSNL1 in CHLA-5 and MB287

A) Zoomed in nodule from CHLA-5: mIHC results from one region of sample CHLA-5. Zoomed in region from Figure 6B stained for Ki67 (yellow), MAP2 (red), and VSNL1 (white). The right side of the image contains as VSNL1+ nodule that is devoid of Ki67+ cycling cells. The left side of the image shows a MAP2+ region with infiltrating Ki67+ cells. Scale bars represent 150 μm.
B) CHLA-5 VSNL1 and Ki67: mIHC and masking results from one patient (CHLA-5). Ki67 and VSNL1 staining patterns for CHLA-5. The rightmost image shows the computationally derived mask for VSNL1+ regions. Scale bars represent 1 mm.
C) MB287 VSNL1 and Ki67: mIHC and masking results from one patient (MB287). Ki67 and VSNL1 staining patterns for MB287. The rightmost image shows the computationally derived mask for VSNL1+ regions. Scale bars represent 1 mm.

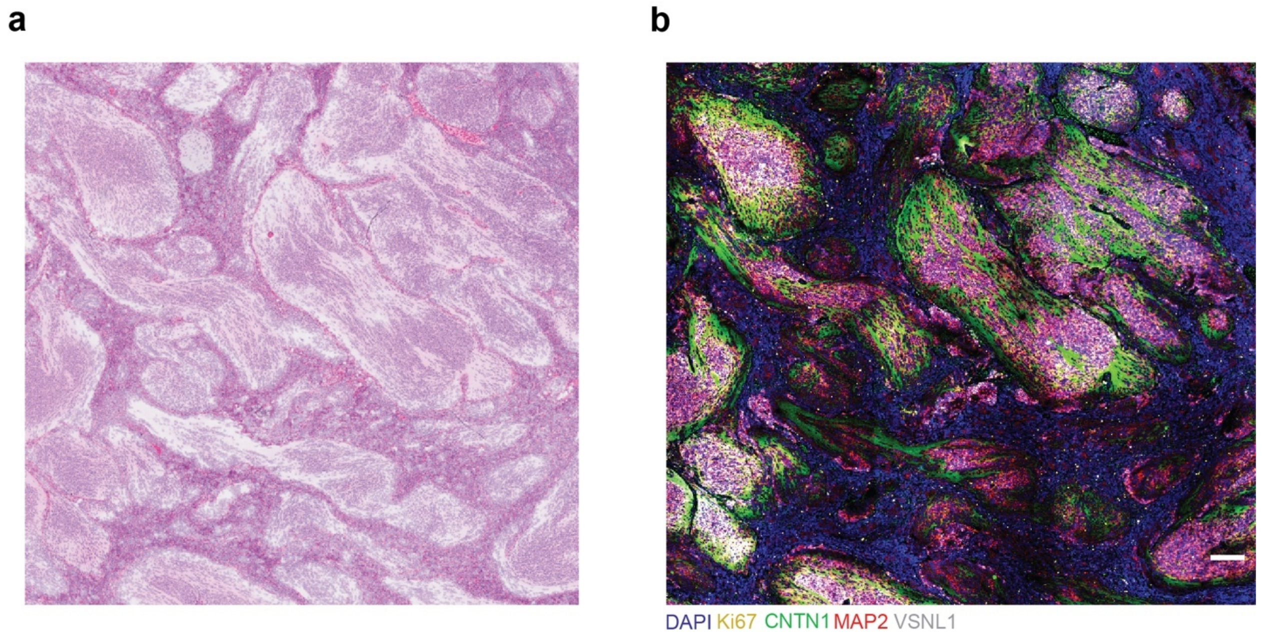

__**Supplementary Figure 21) MBEN Histology Staining for CHLA-10**__
H&E and mIHC results from one section of sample CHLA-10. Left panel shows H&E stain. Right panel shows mIHC staining for DAPI (blue), CNTN1 (green), Ki67 (yellow), MAP2 (red), and VSNL1 (white) for the same region. The abnormal histological structures observed in MBEN tumors show staining consistent with later stages of granule neuron development. Scale bar indicates 100 µm.

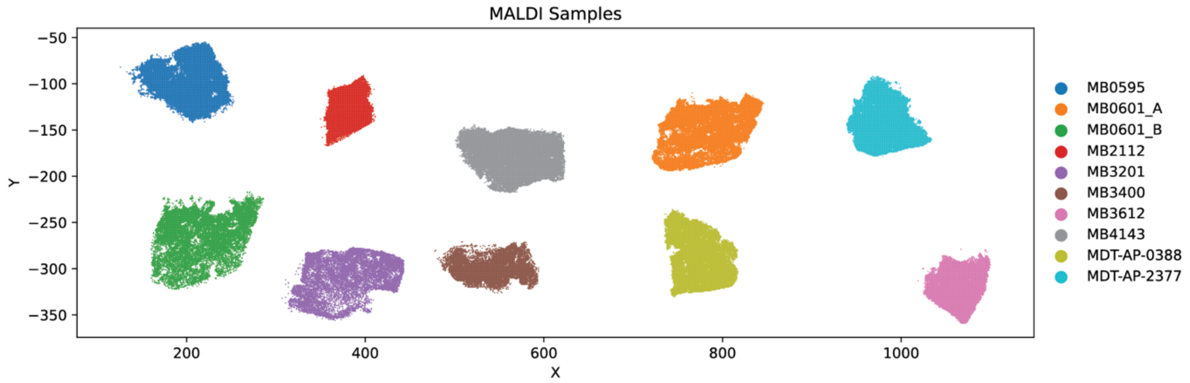

__**Supplementary Figure 22) MALDI Data by Sample**__
Visualization of the high-quality MALDI spots after quality control.

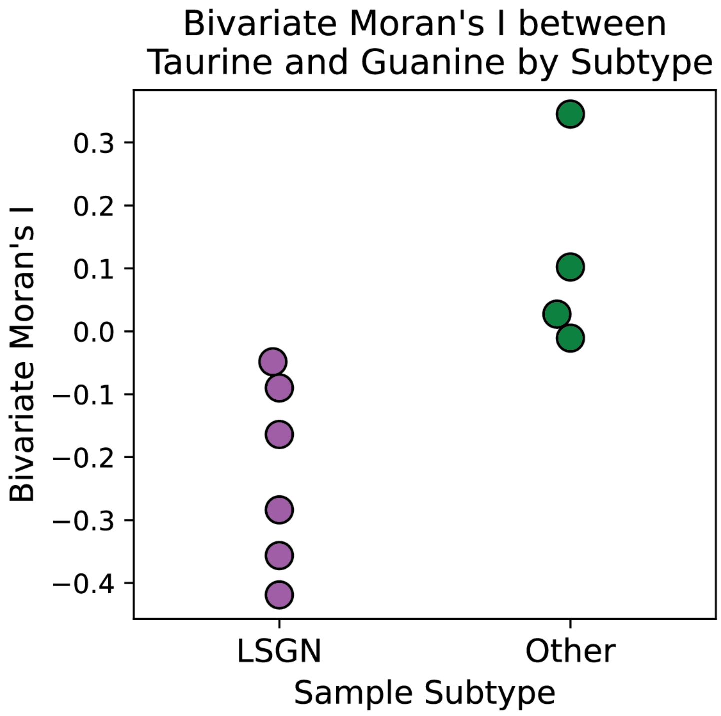

Supplementary Figure 23) Bivariate Moran's I for Guanine and Taurine for Cardinal Data
Replicate of Figure 6B, except processed with an alternate pipeline. The same general trend is observed with negative associations in the tumors with late-stage GNs, while a weakly positive trend appears in the other samples.

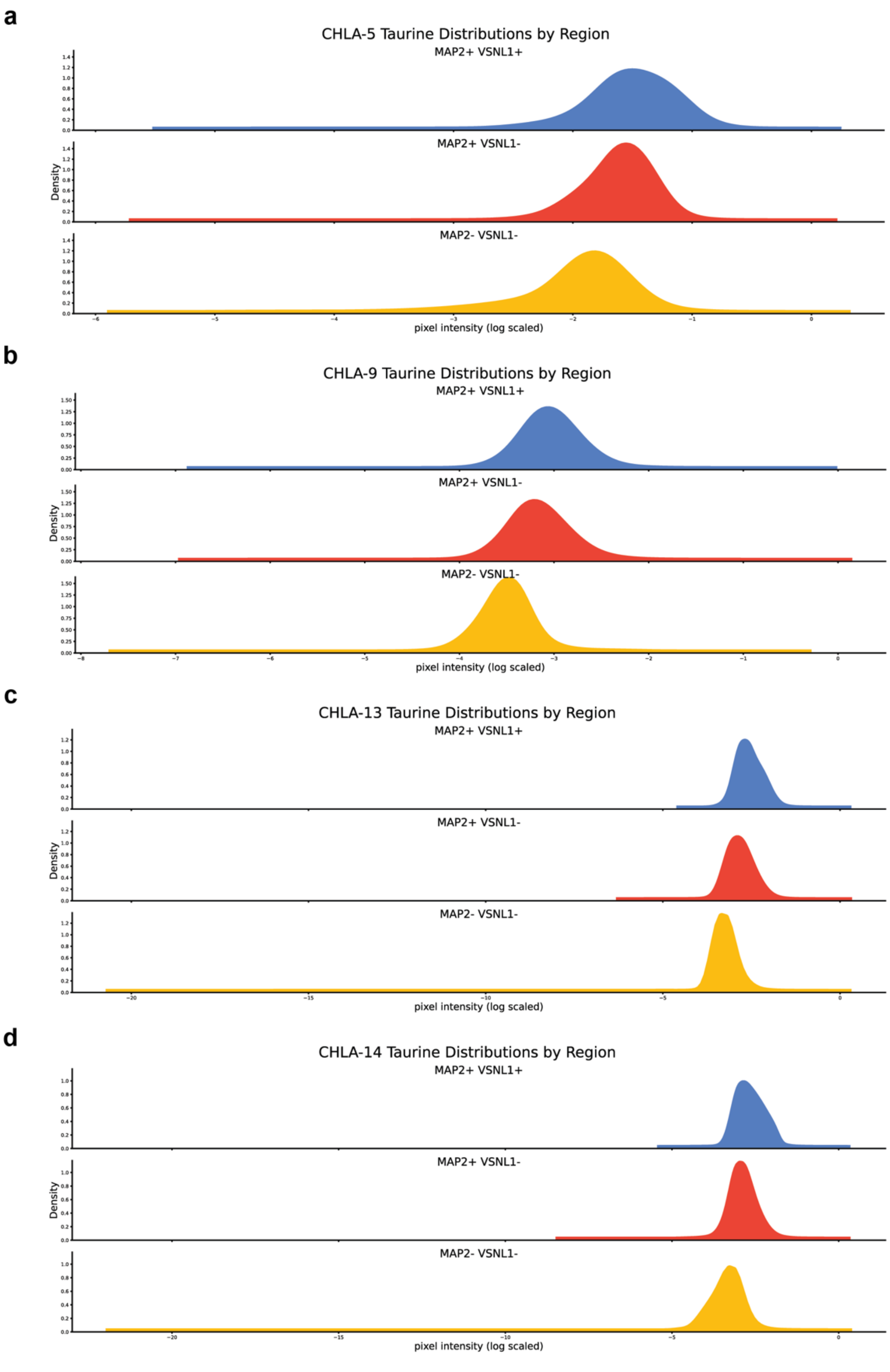

Supplementary Figure 24) Histogram of Taurine Signal MAP2+/VSNL1+, MAP2+/VSNL1-, and MAP2-/VSNL1- Regions Across Samples

Each plot shows the log-normalized fluorescence values from the taurine antibody on the x axis and the density of pixels on the y-axis. The title of every subplot indicates the sample of interest. Within each plot, the individual subplots show the trends for one of the three regions: MAP2+/VSNL1+, MAP2+/VSNL1-, and MAP2-/VSNL1-. A) CHLA-5. B) CHLA-9. C) CHLA-13. D) CHLA-14

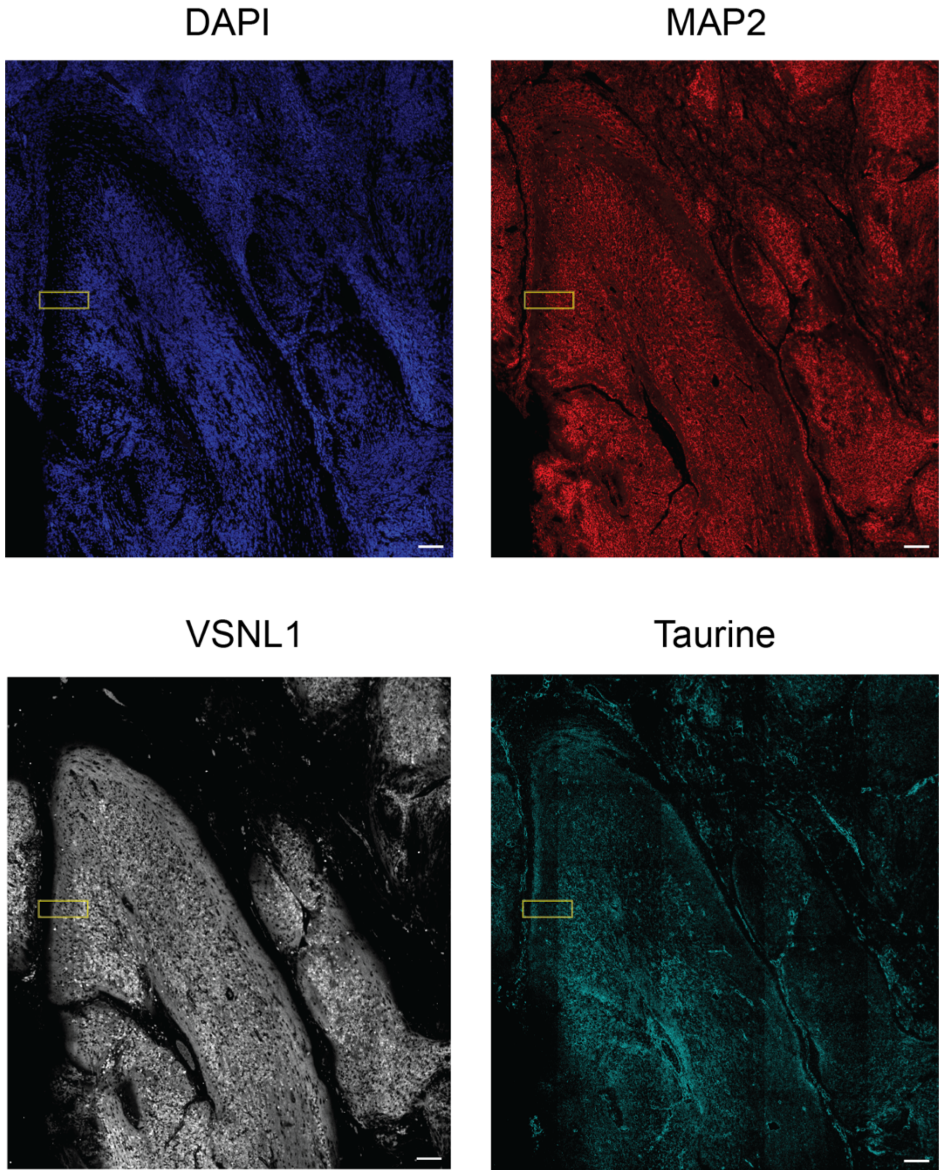

Supplementary Figure 25) Taurine Staining Around Pseudo-Cerebellar Structure in CHLA-10
mIHC results for one region from sample CHLA-10. MAP2 (red), VSNL1 (white), and taurine (cyan). Taurine shows high staining in the internal VSNL1+ regions and at the edges of the cerebellar structure. The yellow box indicates the region selected for Figure 6E. Scale bars indicate 100 μm.

Antigen: **CNTN1**
Tissue: Cerebellum
Dilution: 15 ug/ml
Host: Goat
Manufacturer: Novus
Product Number: AF904
Fluorophore: Directly conjugated with AF-555 after purchase

Antigen: **Ki67**
Tissue: Tonsil
Dilution: 1:50
Host: Rabbit
Manufacturer: Cell Signaling
Product Number: 12075
Fluorophore: Directly conjugated with AF-647 by manufacturer

Antigen: **MAP2**
Tissue: Cerebrum
Dilution: 1:100
Host: Chicken
Manufacturer: Abcam
Product Number: ab92434

Secondary: Goat anti-Chicken IgY (488)
Manufacturer: Invitrogen
Product Number: A32931

Antigen: **Taurine**
Tissue: Small Intestine
Dilution: 1:100
Host: Rabbit
Manufacturer: Sigma-Aldrich
Product Number: AB5022

Secondary: Goat anti-Rabbit IgG (555)
Manufacturer: Invitrogen
Product Number: A32732

Antigen: **VSNL1**
Tissue: Cerebellum
Dilution: 1:100
Host: Mouse
Manufacturer: Invitrogen
Product Number: MA5-26516

Fluorophore: Donkey anti-Mouse IgG (555)
Manufacturer: Invitrogen
Product Number: A32773

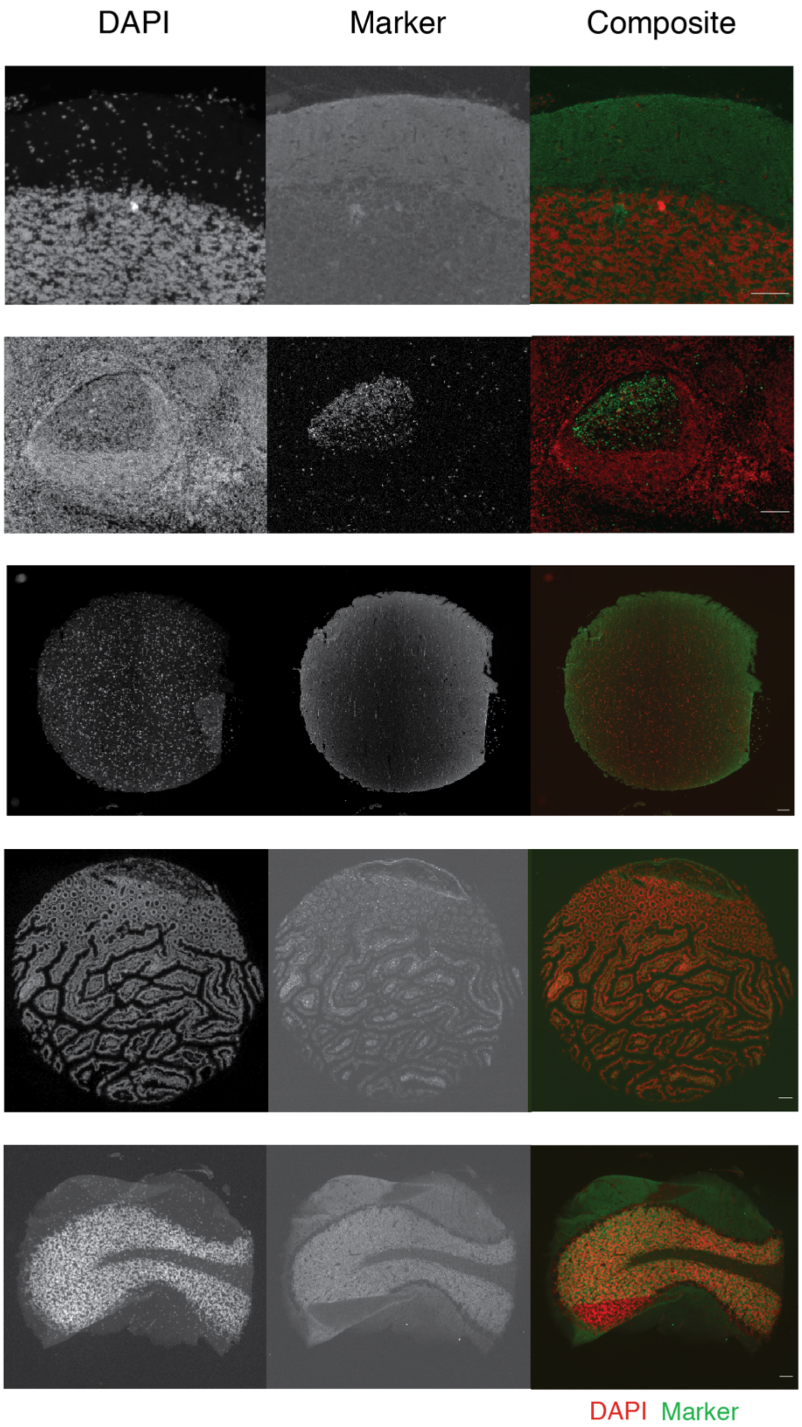

Supplementary Figure 26) Antibody Validations
Antibody details and validation images for CNTN1, Ki67, MAP2, taurine, and VSNL1. Each row shows staining for an individual sample. The first column shows DAPI fluorescence, and the second column shows staining for the marker. The third column is a composite with DAPI in red and the marker of interest in green. Scale bars are in the rightmost image of each row and indicate 100μm.

VSNL1

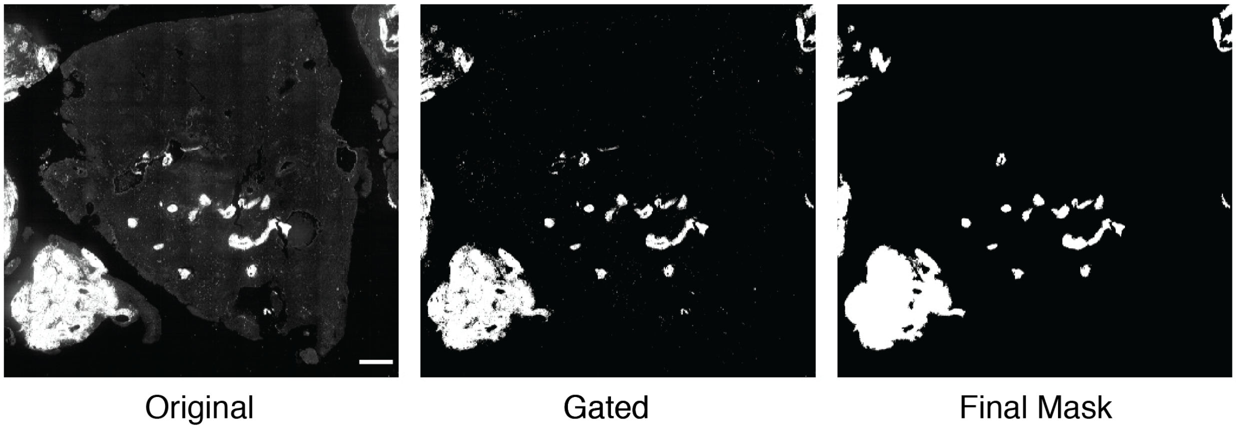

Supplementary Figure 27) Masking Strategy for VSNL1 in CHLA-5

VSNL1 staining for one sample (CHLA-5). The left image indicates the log-normalized intensities for the VSNL1. The middle panel shows the pixels above the gating threshold. Finally, the right panel shows the final mask generated to indicate continuous regions of positive signal for each marker. Scale bars indicate 1 mm.

Supplementary References

1. Archer, T. C. *et al.* Proteomics, Post-translational Modifications, and Integrative Analyses Reveal Molecular Heterogeneity within Medulloblastoma Subgroups. *Cancer Cell* (2018) doi:10.1016/j.ccell.2018.08.004.
2. Cavalli, F. M. G. *et al.* Intertumoral Heterogeneity within Medulloblastoma Subgroups. *Cancer Cell* (2017) doi:10.1016/j.ccell.2017.05.005.
3. Korshunov, A. *et al.* Transcriptional profiling of medulloblastoma with extensive nodularity (MBEN) reveals two clinically relevant tumor subsets with VSNL1 as potent prognostic marker. *Acta Neuropathol.* (2020) doi:10.1007/s00401-019-02102-z.
4. Okonechnikov, K. *et al.* Mapping pediatric brain tumors to their origins in the developing cerebellum. *bioRxiv* 2021.12.19.473154 (2021) doi:10.1101/2021.12.19.473154.
5. Vladoiu, M. C. *et al.* Childhood cerebellar tumours mirror conserved fetal transcriptional programs. *Nature* (2019) doi:10.1038/s41586-019-1158-7.
6. Riemondy, K. A. *et al.* Neoplastic and immune single-cell transcriptomics define subgroup-specific intra-tumoral heterogeneity of childhood medulloblastoma. *Neuro. Oncol.* (2022) doi:10.1093/neuonc/noab135.
7. Hovestadt, V. *et al.* Resolving medulloblastoma cellular architecture by single-cell genomics. *Nature* (2019) doi:10.1038/s41586-019-1434-6.
8. Darnell, J. C. *et al.* FMRP stalls ribosomal translocation on mRNAs linked to synaptic function and autism. *Cell* (2011) doi:10.1016/j.cell.2011.06.013.
