# Supplementary material for: Developmental basis of SHH medulloblastoma heterogeneity
Source: Nat Commun. 2024 Jan 8;15:270. doi: 10.1038/s41467-023-44300-0 (PMC10774283; doi:10.1038/s41467-023-44300-0)
Supplement: Supplementary file 3 — Description of Additional Supplementary Files [file 41467_2023_44300_MOESM3_ESM.pdf]

Description of Additional Supplementary Files

File Name: Supplementary Data 1
Description: Metadata for samples included in this study

File Name: Supplementary Data 2
Description: Gene signatures used in Figure 2

File Name: Supplementary Data 3
Description: GSVA scores used in Figure 2

File Name: Supplementary Data 4
Description: Differential analysis of GSVA scores across CNVs

File Name: Supplementary Data 5
Description: Metabolite information for MALDI-IMS
