# Supplementary material for: Developmental basis of SHH medulloblastoma heterogeneity
Source: Nat Commun. 2024 Jan 8;15:270. doi: 10.1038/s41467-023-44300-0 (PMC10774283; doi:10.1038/s41467-023-44300-0)
Supplement: Supplementary file 9 — Reporting Summary [file 41467_2023_44300_MOESM9_ESM.pdf]

nature portfolio

Corresponding author(s): Ernest Fraenkel

Last updated by author(s): 12/04/2023

Reporting Summary

Nature Portfolio wishes to improve the reproducibility of the work that we publish. This form provides structure for consistency and transparency in reporting. For further information on Nature Portfolio policies, see our Editorial Policies and the Editorial Policy Checklist.

Statistics

For all statistical analyses, confirm that the following items are present in the figure legend, table legend, main text, or Methods section.

n/a	Confirmed	
☐	☒	The exact sample size (*n*) for each experimental group/condition, given as a discrete number and unit of measurement
☐	☒	A statement on whether measurements were taken from distinct samples or whether the same sample was measured repeatedly
☐	☒	The statistical test(s) used AND whether they are one- or two-sided<br>*Only common tests should be described solely by name; describe more complex techniques in the Methods section.*
☒	☐	A description of all covariates tested
☐	☒	A description of any assumptions or corrections, such as tests of normality and adjustment for multiple comparisons
☐	☒	A full description of the statistical parameters including central tendency (e.g. means) or other basic estimates (e.g. regression coefficient) AND variation (e.g. standard deviation) or associated estimates of uncertainty (e.g. confidence intervals)
☐	☒	For null hypothesis testing, the test statistic (e.g. *F*, *t*, *r*) with confidence intervals, effect sizes, degrees of freedom and *P* value noted<br>*Give P values as exact values whenever suitable.*
☒	☐	For Bayesian analysis, information on the choice of priors and Markov chain Monte Carlo settings
☒	☐	For hierarchical and complex designs, identification of the appropriate level for tests and full reporting of outcomes
☒	☐	Estimates of effect sizes (e.g. Cohen's *d*, Pearson's *r*), indicating how they were calculated

Our web collection on statistics for biologists contains articles on many of the points above.

Software and code

Policy information about availability of computer code

Data collection	Public data collected for this study was downloaded from source databases, as described in the methods section. For newly collected data, snRNA-Seq and scRNA-Seq were performed as described in the methods and raw data processing was performed using 10x Genomics default settings.
Data analysis	For data analysis of scRNA-Seq data, Seurat was used for preprocessing, normalization, and analysis (as described in the methods). All code can be found at https://github.com/fraenkel-lab/shh-mben.<br>The base python code uses the following packages:<br>conda 4.8.3,python: 3.7.8, matplotlib: 3.4.1, numpy: 1.18.5, pandas: 1.3.5, scikit-learn: 0.21.1, scipy: 1.7.3 and seaborn: 0.11.0<br>The MALDI code uses the following packages:<br>conda: 4.7.12, python: 3.8.10, gglasso: 0.1.9, matplotlib: 3.4.2, networkx: 2.6.3, numpy: 1.18.5, pysal: 2.4.0, scanpy: 1.7.2, scikit-learn: 0.24.2, scipy: 1.7.1, seaborn: 0.11.1<br>The R code uses the following packages:<br>R: 4.1.2, biomaRt: 2.50.2, ConsensusClusterPlus: 1.58.0, data.table: 1.14.2, ggplot2: 3.3.5, gridExtra: 2.3, GSVA: 1.42.0, harmony: 0.1.0, monocle3: 1.0.0, RColorBrewer: 1.1.2, readxl: 1.3.1, Seurat: 4.1.0, SeuratWrappers: 0.3.0, stringr: 1.4.0

For manuscripts utilizing custom algorithms or software that are central to the research but not yet described in published literature, software must be made available to editors and reviewers. We strongly encourage code deposition in a community repository (e.g. GitHub). See the Nature Portfolio guidelines for submitting code & software for further information.

Data

Policy information about availability of data

All manuscripts must include a data availability statement. This statement should provide the following information, where applicable:

- Accession codes, unique identifiers, or web links for publicly available datasets
- A description of any restrictions on data availability
- For clinical datasets or third party data, please ensure that the statement adheres to our policy

The scRNA-seq and snRNA-seq data generated for this manuscript have been deposited in GEO under the accession number GSE214469 [https://www.ncbi.nlm.nih.gov/geo/query/acc.cgi?acc=GSE214469]. Previously published data used for this work can be accessed through the following GEO accession numbers: scRNA-seq data from Riemondy et al. 8 GSE156053 [https://www.ncbi.nlm.nih.gov/geo/query/acc.cgi?acc= GSE156053], scRNA-seq data from Hovestadt et al. 7 GSE119926 [https://www.ncbi.nlm.nih.gov/geo/query/acc.cgi?acc= GSE119926], scRNA-seq data from Vladoiu et al. 6 GSE118068 [https://www.ncbi.nlm.nih.gov/geo/query/acc.cgi?acc= GSE118068], and bulk RNA transcriptomics from Cavalli et al. 5 GSE85218 [https://www.ncbi.nlm.nih.gov/geo/query/acc.cgi?acc= GSE85218].

mIHC imaging data can be found at https://zenodo.org/records/10257144 and taurine-focused IHC imaging can be found at https://zenodo.org/records/10256482. Processed omics data can be found with the corresponding code at https://github.com/fraenkel-lab/shh-mben. The remaining data are available within the Article, Supplementary Information, or the Source Data file.

Research involving human participants, their data, or biological material

Policy information about studies with human participants or human data. See also policy information about sex, gender (identity/presentation), and sexual orientation and race, ethnicity and racism.

Reporting on sex and gender	Sex and gender information are not considered for this study because of the scarcity of tumor tissue and our focus on histological variability.
Reporting on race, ethnicity, or other socially relevant groupings	Race and ethnicity are not considered for this study because of the scarcity of tumor tissue and our focus on histological variability.
Population characteristics	Population characteristics are not considered for this study.
Recruitment	This study focuses on tumors SHH medulloblastoma tumors with MBEN histology. Tumor tissue from consenting patients with this histological designation were chosen for snRNA-seq analysis. snRNA-seq analysis of tumors with known proteomic subtypes were chosen based on their proteomic subtype status determined from bulk proteomics studies. FFPE slides from samples used for multiplexed immunohistochemistry were chosen based on their molecular subtype (SHH).
Ethics oversight	All experiments in this study involving human tissue or data were conducted in accordance with the Declaration of Helsinki. All tissues used in this study were obtained with properly informed written consent of patients or their legal representatives.<br><br>For MBEN single-cell transcriptomics analysis, all collection and experimental procedures were performed after approval by the institutional review board at The Hospital for Sick Children (Toronto, Canada). For snRNA-seq of samples with known proteomic subtypes, this study was approved by the Institutional Clinical Research Board of Gustave Roussy, and complied with the reference methodology MR-004 (IRB number : 2022-125). For mIHC and imaging analysis of samples from CHLA, all samples were deidentified and obtained with properly informed consent. This study was approved by the Institutional Review Board at Children's Hospital Los Angeles (CHLA-20-00588). FFPE material for the other samples used for mIHC analysis was obtained with informed consent based on the International Cancer Genome Consortium (ICGC) guidelines. This was approved by the Ethics Committee of the Medical Faculty at Heidelberg University and by the Institutional Review Board of Contributing Center Nikolay Nilovich Burdenko Neurosurgical Institute in Moscow.

Note that full information on the approval of the study protocol must also be provided in the manuscript.

Field-specific reporting

Please select the one below that is the best fit for your research. If you are not sure, read the appropriate sections before making your selection.

[x] Life sciences [ ] Behavioural & social sciences [ ] Ecological, evolutionary & environmental sciences

For a reference copy of the document with all sections, see nature.com/documents/nr-reporting-summary-flat.pdf

Life sciences study design

All studies must disclose on these points even when the disclosure is negative.

Sample size	Due to the rarity of MB tumors, we did not use a statistical method to determine sample size. We collected single-nucleus RNA-sequencing

	data from 13 patients and single-cell RNA-sequencing data from 1 patient. 8 samples were chosen because they have the histological designation "medulloblastoma with extensive nodularity" (MBEN). The other 6 were chosen because they have known SHHa/SHHb protein subtype status. In total, there are only 14 SHH MB tumors with published scRNA-seq data. This study alone has 13 individual tumors and should provide more power than the prior studies to detect variability between samples and evaluate trends on the histological subtype of interest.
Data exclusions	No samples were explicitly excluded from this study. Individual cells were excluded when they did not meet quality control criteria (see methods). In some instances, specific clusters of cells were excluded from downstream analyses because they were only observed in single patients.
Replication	We observed our initial trend on 7 tumors with MBEN histology. We performed snRNA-seq on an additional 6 tumors with other histological designations and found that the general trends can be replicated and are not just specific to MBEN histology. Additionally, Ghasemi et al found the same high-level trends on an independent cohort of tumors. Given the precious nature of these rare tumor samples, technical replicates were not used for scRNA-seq or imaging studies.
Randomization	There was no explicit randomization for this study. MBEN is a histological subtype of a rare brain tumor, so any tumors found that meet this criteria with enough tissue for sequencing were chosen for the study.
Blinding	The study did not make use of clinical outcome data, so blinding was not relevant.

Reporting for specific materials, systems and methods

We require information from authors about some types of materials, experimental systems and methods used in many studies. Here, indicate whether each material, system or method listed is relevant to your study. If you are not sure if a list item applies to your research, read the appropriate section before selecting a response.

Materials & experimental systems

n/a	Involved in the study
☐	☒ Antibodies
☒	☐ Eukaryotic cell lines
☒	☐ Palaeontology and archaeology
☒	☐ Animals and other organisms
☒	☐ Clinical data
☒	☐ Dual use research of concern
☒	☐ Plants

Methods

n/a	Involved in the study
☒	☐ ChIP-seq
☒	☐ Flow cytometry
☒	☐ MRI-based neuroimaging

Antibodies

Antibodies used	All antibodies used were described in Supplementary Figure 26.<br>CNTN1: Novus-AF904 (15µg/ml), directly conjugated with AF-555 after purchase<br>Ki67: Cell Signaling 12075 (1:50), directly conjugated AF-647 by manufacturer<br>MAP2: abcam ab92434 (1:100). secondary antibody is Invitrogen A32931 (Goat Anti-Chicken)<br>Taurine: Sigma-Aldrich AB5022 (1:100). Secondary antibody is Invitrogen A32732 (Goat Anti-Rabbit)<br>VSNL1: Invitrogen MA5-26516 (1:100). Secondary antibody is Invitrogen A32773 (Donkey Anti-Mouse)
Validation	Each antibody has been previously published and was validated again by testing the antibody on FFPE from human tissue where the marker is known to be expressed (Supplementary Figure 26).<br>CNTN1: cerebellum<br>Ki67: tonsil<br>MAP2: cerebrum<br>Taurine: small intestine<br>VSNL1: cerebellum<br><br>Further information about the primary anitbodies can be found at their source websites listed below:<br>CNTN1: https://www.novusbio.com/products/contactin-1-antibody_af904<br>Ki67: https://www.cellsignal.com/products/antibody-conjugates/ki-67-d3b5-rabbit-mab-alexa-fluor-647-conjugate/12075<br>MAP2: https://www.abcam.com/products/primary-antibodies/map2-antibody-ab92434.html<br>Taurine: https://www.sigmaaldrich.com/US/en/product/mm/ab5022<br>VSNL1: https://www.fishersci.com/shop/products/vsnl1-monoclonal-antibody-oti4a6-invitrogen/PIMA526516
